# Uptake of Australia’s Health Star Rating System

**DOI:** 10.3390/nu10080997

**Published:** 2018-07-30

**Authors:** Alexandra Jones, Maria Shahid, Bruce Neal

**Affiliations:** 1 The George Institute for Global Health, UNSW, Sydney, NSW 2042, Australia; mshahid@georgeinstitute.org.au (M.S.); bneal@georgeinstitute.org.au (B.N.); 2 Charles Perkins Centre, University of Sydney, Sydney, NSW 2006, Australia; 3 Department of Epidemiology and Biostatistics, School of Public Health, Faculty of Medicine, Imperial College London, London SW7 2AZ, UK

**Keywords:** front-of-pack, food labelling, health star rating, nutrient profiling

## Abstract

In June 2014, Australia and New Zealand adopted a voluntary front-of-pack nutrition labelling scheme in the form of the Health Star Rating (HSR) system. Our aim was to assess its uptake in Australia while a formal five-year review of the system is underway. Numbers and proportions of products eligible to carry a HSR were recorded each year between 2014 and 2017 as part of an annual survey of four large Australian retail outlets. Mean HSR values were determined for products that were and were not labelled with a HSR logo, and summary data presented overall, by HSR score, by major food category, and for leading manufacturers. Results show that uptake is increasing: HSR appeared on 4348/15,767 (28%) of eligible products in 2017 and has now appeared on 7922 products since implementation. Of those products displaying a HSR logo, more than three-quarters (76.4%) displayed a HSR of ≥3.0. Products displaying a HSR logo had a higher mean HSR (3.4), compared to products not displaying a HSR logo (2.7). Uptake was highest on convenience foods (44%), cereals (36.7%), and fruit and vegetable products (35.9%). More than 100 manufacturers were using the system, but retailers Coles, Woolworths and Aldi were together responsible for 54% of uptake. For all except Coles, Woolworths and Campbell Arnott’s, the mean HSR of products displaying a logo on pack was higher than products made by that manufacturer not showing a HSR logo. We conclude that to ensure the consistent and widespread uptake required for consumers to make informed food purchases, HSR should be made mandatory at the conclusion of the five-year review.

## 1. Introduction

Interpretive front-of-pack nutrition labels (FoPL) are recommended by the World Health Organization (WHO) as an evidence-based policy to promote healthier diets [1,2]. These types of labels use nutrient profiling to assess the nutritional quality of individual foods and display this in a simplified, graphical form on the front-of-pack. There is growing evidence that FoPL have potential to improve nutrition literacy, guide consumer choice, and incentivize industry to improve their formulations [3,4,5]. While not a complete source of dietary advice, FoPL is recognized as a helpful tool to use in conjunction with complementary interventions such as food-based dietary guidelines aimed at improving the overall nutritional quality of diets [6].

In June 2014, following a lengthy process of development involving federal, state and territory governments in collaboration with industry, public health and consumer groups, Australia and New Zealand adopted a voluntary FoPL: The Health Star Rating (HSR) system [7]. In short, its purpose is to ‘provide convenient, relevant and readily understood nutrition information and/or guidance on food packs to assist consumers to make informed food purchases and healthier eating choices’ [8].

The HSR system comprises three components: The HSR algorithm, the HSR graphic, and an accompanying education campaign [9]. The HSR algorithm was developed by Food Standards Australia New Zealand (FSANZ) in consultation with technical and nutrition experts, including industry representation [10]. It is adapted from an existing model (Nutrient Profiling Scoring Criteria, NPSC) used to regulate eligibility to display health and nutrient content claims in both countries, contained within the Australia New Zealand Food Standards Code [7]. The NPSC itself was derived from the validated United Kingdom Ofcom model used to regulate marketing of foods to children in the UK [8,11,12].

The HSR algorithm generates a star rating from 0.5 (least healthy) to 5.0 stars (most healthy) in ten half-star increments. Ratings are determined by an overall assessment of ‘risk’ components (total energy, total sugars, saturated fat, sodium) that align with those nutrients recommended to be limited in the dietary guidelines of both countries, and ‘positive’ components of food (protein, fiber and fruit, vegetable, nut and legume content (FVNL)). HSR values determined from the algorithm align well overall with available definitions of healthy (core) and unhealthy (discretionary) foods contained within the Australian Dietary Guidelines (ADGs) [13,14,15].

Where they elect to display a HSR, food manufacturers are responsible for correct and accurate use according to government-issued guidance [16,17]. A number of variants of the HSR label are permitted [17] (Appendix A). Where the label is being used, monitoring suggests the vast majority of manufacturers are compliant with the HSR Style Guide [18] though the voluntary nature of the program may be resulting in the selective application of HSRs to foods with better nutrient profiles, including those classified ‘discretionary’ by the ADGs [19].

At its adoption, Australian and New Zealand Food Ministers agreed HSR would remain voluntary for five years, and subject to a two-year review of progress [20]. They later agreed to a comprehensive formal review, due to be delivered by mid-2019 [21]. With that review underway, our objective is to report on the progress of HSR implementation in Australia in its fourth year.

## 2. Materials and Methods

The primary analysis of HSR uptake comprises between-year comparisons of serial cross-sectional surveys of the packaged foods available in selected Australian supermarkets from 2014 to 2017. The secondary analyses are cross sectional examinations of the combined data from these surveys supplemented with information about additional products derived from other sources.

### 2.1. Data Source

The George Institute for Global Health uses its FoodSwitch system to collect information about the food supply in Australia and multiple other jurisdictions. Primary data about packaged foods are extracted directly from food packaging with secondary variables derived as required. The Australian FoodSwitch program maintains two databases that have been used for these analyses:

1. The Australian FoodSwitch Monitoring Database comprises annually updated information sourced by trained data collectors through in-store surveys conducted annually at the same four supermarkets in metropolitan Sydney (one Coles, Woolworths, Aldi and IGA). Images of the food packaging are captured (front of pack, nutrient declaration, ingredients list, manufacturer details), using a bespoke smartphone application and then the data are extracted and the images stored. The database is designed specifically to facilitate longitudinal analysis of trends in the Australian packaged food supply. We used a Monitoring Database extract covering 2014, 2015, 2016 and 2017 to compare annual uptake of HSR labelling on pack since HSR program inception.
2. The Australian FoodSwitch Full Database incorporates information about all products included in the Monitoring Database, together with additional product information either provided directly by the food industry or sourced through crowd-sourcing via the FoodSwitch smartphone application [22]. We used a Full Database extract for the period 30 June 2014 to 30 June 2018 to estimate the total number of products that have carried a HSR at any point since the system’s introduction across the Australian food supply.

### 2.2. Food Labelling and Food Composition Data

The presence or absence of HSR labelling has been routinely determined at data entry since 2015 by examining images of product labels. If HSR labelling was being used, we recorded whether the label was the full HSR logo (Appendix A: Options 1–4), or the permitted energy icon only (Appendix A: Option 5). Where a HSR logo was present, we recorded the HSR value displayed (from 0.5 to 5.0). The presence of HSR labelling was not routinely recorded in 2014 in light of HSR’s then recent introduction and is therefore taken as zero for this report.

For all products we also extracted information from the nutrition information panel on back of pack. Energy (kJ/100 g), protein (g/100 g), saturated fat (g/100 g), total sugar (g/100 g), and sodium (mg/100 g) are mandatory on the Australian nutrient declaration but details on FVNL (%), concentrated FVNL (%), and fiber (g/100 g) are optional. Where such details were not provided by the manufacturer on the package, appropriate levels were estimated using information drawn from the back-of-pack ingredients list, generic food composition databases, or by analogy with similar products using methods described previously [22]. The estimation process provides a proxy value for each nutritional indicator at the finest category level for more than 1000 individual food subcategories. Proxy values are then substituted for each product in that category for which data are missing.

We also extracted the manufacturer of each product.

### 2.3. Product Categorization and Eligibility for HSR

Categorization of products was based on the system developed by the Global Food Monitoring Group and incorporated into FoodSwitch [23]. This hierarchical system is designed to monitor the nutrient composition of processed foods around the world. It classifies foods into major categories (e.g., bread and bakery products), minor categories (e.g., bread; biscuits), and subcategories (e.g., savory biscuits; sweet filled biscuits).

Our analysis included only packaged food items. We excluded infant foods and formula, vitamins and supplements, formulated supplementary sports foods, foods for special medical purposes and alcoholic beverages because these foods have been specifically deemed outside the scope of the HSR system [17]. This left 15 major categories for analysis. Within these, we also excluded subcategories of plain tea and coffee, herbs and spices, baking powders, yeasts and gelatins, as these foods do not contribute significantly to nutrient intake, are not required to display a nutrition information panel [24], and are also therefore not required to display a HSR.

Products were identified by their unique barcode. Where the same product (i.e., same item, in same product size), appeared in more than one store surveyed, we counted it only once. Where a product appeared in more than one package size (i.e., had a different barcode), each package size was counted as an individual product. This approach captures the number of product packages that have been updated by manufacturers to display HSR.

### 2.4. Calculation of the Health Star Rating

Where a product was displaying a HSR logo on its label, we used the HSR value displayed by the manufacturer for the purpose of our analysis.

In cases where a HSR logo was not being displayed (either because the manufacturer had not adopted the HSR system, or had elected to display the energy icon variant only), a HSR was calculated in alignment with the methods described in the ‘Guide for Industry to the Health Star Rating Calculator’ [16]. In short, foods were categorized into one of six HSR categories (i.e., non-dairy beverages; dairy beverages; oils and spreads; cheese and processed cheese; all other dairy foods; all other non-dairy foods). Baseline points were calculated based on the energy, saturated fat, total sugar, and sodium content per 100 g. Modifying points for FVNL%, concentrated FVNL%, protein, and fiber were calculated, where applicable. A HSR ‘score’ was calculated by subtracting the modifying points from baseline points. This score is then converted to a HSR based upon a defined scoring matrix for each of the six categories. The HSR ranges from 0.5 to 5.0 stars in ten half-star increments. A higher HSR reflects a healthier product.

### 2.5. Statistical Analysis

For the primary analysis, HSR uptake was determined separately for each year by dividing the number of products carrying the HSR logo or energy icon variant by the total number of eligible products, to obtain the percentage uptake of HSR. Analysis was done using data for 2014 to 2017 derived from the FoodSwitch Monitoring Database. We also made an additional overall estimate of uptake with the same approach using the FoodSwitch Full Database for products captured at any time between 30 June 2014 up to and including 30 June 2018.

Based upon the FoodSwitch Monitoring Database 2017 extract, we also determined the proportions of products displaying HSR by each HSR value 0.5–5.0, by 15 major food categories, and for manufacturers with at least 100 products eligible to display HSR. In each case the mean HSR of products displaying the HSR logo was compared against the mean of all products eligible to carry the HSR but not displaying a logo, either because they do not use HSR at all, or use the energy icon only.

A list of all manufacturers captured using the HSR system on at least one product in the Monitoring Database 2017 is also included at Appendix B.

## 3. Results

### 3.1. HSR Uptake over Time

**Figure 1:**
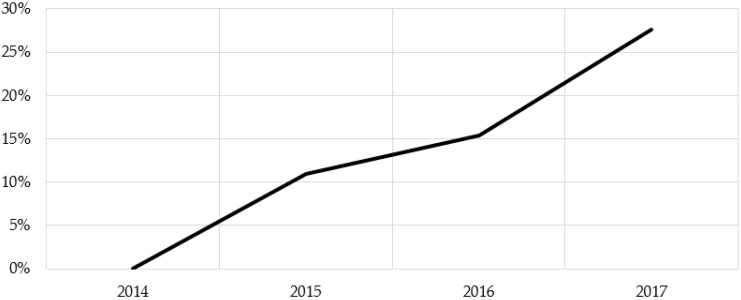
Health Star Rating (HSR) system uptake by proportion of HSR eligible products displaying the HSR logo or permitted energy icon in the FoodSwitch Monitoring Database.

Within the FoodSwitch Monitoring Database in 2017 there were 4348 products using the HSR system out of 15,767 eligible products. Of these, 3755 were displaying the HSR logo, and 593 the energy icon only. Together these products represented 28% of all HSR eligible products and suggest an approximately linear increase in products using HSR each year since the system was introduced (Figure 1).

There were 7922 products in the FoodSwitch Full Database using HSR, representing 14% of the 54,798 HSR eligible products captured between 30 June 2014 and 30 June 2018.

### 3.2. HSR Uptake by HSR Value

**Figure 2:**
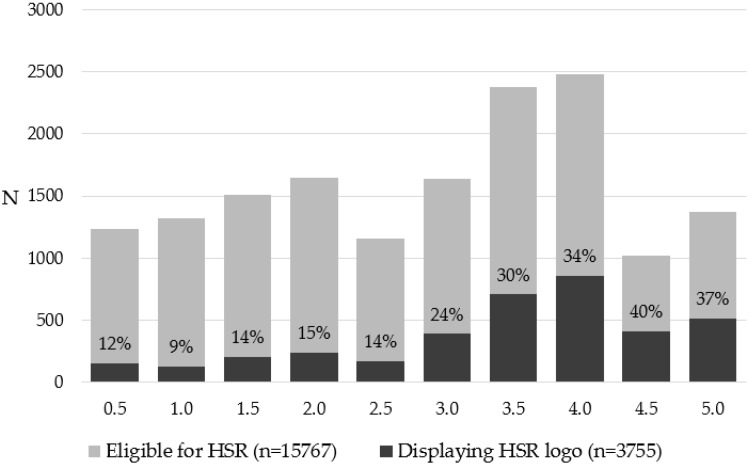
Uptake of the HSR logo by HSR value in the 2017 FoodSwitch Monitoring Database. Percent numbers indicate proportion of eligible products with each HSR value that carry a HSR logo on pack.

Products receiving a higher HSR were more likely to use the HSR logo (Figure 2). HSR 4.0 had the highest number of products displaying a HSR logo. Proportionate uptake was lowest on products that would receive HSR 1.0, where only 9% of eligible products were using the logo. By contrast, 40% of products eligible to display HSR 4.5 were displaying the logo. Of those products displaying a HSR logo, 2870 (76.4%) displayed a HSR of ≥3.0.

### 3.3. HSR Uptake by Category

Uptake of HSR varied by category, with convenience foods (44.4%), cereal and grain products (36.7%), and fruit and vegetable products (35.9%) having the highest uptake, and eggs (12.5%), sugars, honey and related products (13.8%), and sauces, dressings, spreads and dips (14.2%) having the lowest proportion of uptake (Table 1).

In all categories except sugars, honey and related products, and eggs, the mean HSR of products displaying a HSR logo was higher than the mean of those products not using the HSR system, or displaying the energy icon only. This was most pronounced in non-alcoholic beverages, where the mean was HSR 4.3 for products carrying a HSR logo, and HSR 2.5 for those without.

Of the 591 products using the energy icon only, 415 (70.2%) were from the confectionery and non-alcoholic beverage categories. Over three quarters (77.5%) of products using the energy icon would receive a HSR between 0.5–2.0.

### 3.4. Uptake by Manufacturer

Table 2 sets out HSR uptake among manufacturers. Retailers Coles, Woolworths and Aldi were cumulatively responsible for over half (54%) of all products using HSR, and Coles displayed HSR on over 1000 products. For all except Coles, Woolworths and Campbell Arnott’s, the mean HSR of products displaying a HSR logo on pack was higher than products made by that manufacturer not showing a HSR logo. Results are listed individually for manufacturers with >100 HSR eligible products. A supplementary list of the 118 manufacturers captured applying HSR to at least one product appears at Appendix B.

## 4. Discussion

Four years since implementation commenced, voluntary uptake of HSR is increasing but remains modest and uneven, limiting its public health impact.

These independent findings from the FoodSwitch Monitoring Dataset are comparable to government-commissioned annual monitoring done by the Heart Foundation, which last reported HSR on 3580 products (349 of which displayed the energy icon only) in June 2017 [25]. Heart Foundation monitoring comprises systematic collection over four consecutive weeks in several large supermarkets in metropolitan Victoria but reporting did not include a denominator, so a comparable estimate of proportionate uptake in 2017 was not possible.

The 7922 products in the FoodSwitch Full Database reported to have displayed an HSR since 2014 represents uptake for current and delisted products. The figure can be compared to government statements made in June 2018 suggesting more than 10,300 products have now displayed HSR [26]. The discrepancy between the two numbers may reflect additional data provided direct to government by the food industry, seasonal products not systematically captured by the FoodSwitch database and products where artwork has been reported as updated to government but for which new labels have not yet been placed on shelf stock. Government figures also include data from online searches, which are not part of the FoodSwitch data collection process. While the FoodSwitch figure represents 14% of all HSR eligible products in the database, government does not provide a denominator of eligible products, nor an estimate of proportionate uptake.

We found 118 manufacturers using HSR in 2017 in the four large stores included in the Monitoring Dataset, whereas the HSR website contains a list of 169 companies (manufacturers and in some cases brands) who self-report implementing HSR [9].

HSR uptake can be compared to the implementation of other food labelling initiatives overseas and in Australia. For example, the United Kingdom’s voluntary traffic light front-of-pack nutrition label (TLL) has been implemented since 2013, with estimates suggesting retailers and manufacturers that display TLL account for >60% of packaged foods sold [27]. In Australia, HSR uptake can be compared with that of the industry-led Daily Intake Guide (DIG), which appeared on over 7200 products in all major categories at its last audit by the Australian Food and Grocery Council (AFGC) in 2014 [28]. With evidence suggesting the DIG is the least well-performing of the series of front-of-pack labels [29,30,31], it is now time the AFGC updated its best practice guidance to members [32], committing fully to HSR and ensuring the DIG is removed from the marketplace.

Voluntary approaches to front-of-pack nutrition labelling can be contrasted with recent implementation of mandatory Country of Origin Labelling (CoOL) on packaged foods in Australia [33]. Adopted in 2016, regulations provide a two-year transition period, with compliance required by 1 July 2018, demonstrating the feasibility of widespread compulsory labelling changes when driven by sufficient political will.

The differential use of the HSR logo on products at the upper end of the HSR spectrum is unsurprising given the voluntary nature of the system but confirms the perception that some companies are using HSR primarily as a marketing tool [19]. The HSR Style Guide specifically encourages food companies adopting the system ‘to do so consistently across their product range and/or within product categories’ [17]. The major Australian retailers have demonstrated clear leadership in this regard and it is encouraging that HSR uptake is highest in several food categories where consumers report being most likely to use nutrition labelling, including cereals and pre-prepared meals [34]. However, uptake across and within categories remains uneven. Previous analysis has demonstrated the vast majority of products scoring HSR ≤2.0 are classified as discretionary or ‘junk’ foods [13], recommended to be limited in the Australian Dietary Guidelines but currently responsible for one third of adults’ and almost 40% of Australian children’s energy intake [35]. Uptake of the HSR logo on these products remains very low.

During HSR’s development in 2013, State and Federal food ministers agreed that HSR would remain voluntary subject to there being consistent and widespread uptake. At that time they suggested that if voluntary implementation was found to be unsuccessful, a mandatory approach would be required [36]. Now four years into HSR’s implementation, our results suggest consistent and widespread uptake has still not been achieved. Application of the HSR logo to all products, regardless of healthiness, is key to transparency and will be vital for informing and guiding consumer choices.

Our results also highlight significant use of the energy icon-only variant of the HSR system. While reports on how the energy icon was intended by HSR’s developers to be used vary [10,37], internal beverage industry documents suggest that in that category its adoption is being used as a strategic ‘shield’ to avoid stronger forms of interpretive labelling [38]. HSR’s government-issued Style Guide places the energy icon last among a ‘hierarchy’ of options, suggesting that it ‘may be used alone (e.g., on small packages where the full HSR system graphic could not be accommodated)’ [17]. Our results suggest broader use on confectionery, and selective application to non-alcoholic beverages that receive a low HSR. In light of its similarity to the DIG, this variant of the label is likely to be of limited utility to consumers, suggesting its use should be more tightly restricted to maximize the impact of the HSR system. For consistency of consumer use, all HSR eligible products should display a HSR logo except those genuinely restricted by package size as suggested in the Style Guide. While previous research has demonstrated the current HSR algorithm derives appropriate values for the vast majority of products [13], the current five year review provides opportunity for refinements to further support consistent use of the full HSR logo across the food supply.

The present report benefits from the use of multiple datasets and complementary analyses. The Monitoring Dataset is robust for time trends but is weak for absolute coverage of the overall food supply, given its reliance upon four metropolitan stores. By contrast, the Full Dataset provides better coverage of the full food supply, but may include discontinued and seasonal products, and constancy of data collection methods over time cannot be assured. HSR uptake for 2014 was estimated as zero given the absence of systematic collection of HSR data at this point in time and it is likely that there were a small number of products displaying HSR logos by the end of 2014. Where a HSR was provided by a manufacturer we used this in our analysis, but where a HSR logo was not present on the label it was necessary to generate a HSR. As FVNL content and fiber are not currently mandatory on back-of-pack nutrition information panels in Australia, missing values were therefore estimated from ingredients lists, food composition databases, and other sources. It is possible that the presence of HSR logos on some food labels may have been missed at the time of data entry and this also could lead to under-estimation of the uptake of HSR labelling. While the HSR algorithm itself is not the focus of this paper, we note that both the HSR algorithm, and the UK Ofcom model on which it is derived, are both currently under review and may be subject to updates that could impact HSR scores received by products [39,40].

## 5. Conclusions

Uptake of the HSR system in its first four years is promising, but far from consistent and widespread. The formal five-year review provides an ideal opportunity to address concerns with HSR’s operation, including work already underway to strengthen performance of the HSR algorithm, and the need for greater clarity around the use of the energy icon variant suggested by our results. Once the review concludes in 2019, HSR should be made mandatory to maximize its public health impact in assisting consumers to make informed food purchases and healthier eating choices.

## Figures and Tables

**Table 1:** HSR uptake by food category and mean HSR by food category among products displaying the HSR logo and among products either not displaying the HSR logo or carrying the energy icon only, in the 2017 FoodSwitch Monitoring Dataset.

Category	Products Surveyed	Displaying HSR/Eligible to Display HSR	Displaying HSR	Mean HSR	
	*N*	*n*/*N*	%	HSR Logo on Pack	No HSR/Energy Icon Only
Bread and bakery products	1922	472/1765	26.7	2.3	2.2
Cereal and grain products	1771	634/1728	36.7	4.0	3.4
Confectionery	1152	290/1010	28.7	1.6	1.3
Convenience foods	1238	525/1182	44.4	3.5	3.3
Dairy	2274	437/2176	20.1	3.0	2.7
Edible oils and oil emulsions	351	63/346	18.2	3.0	2.5
Eggs	64	8/64	12.5	4.0	4.0
Fish and fish products	604	171/596	28.7	3.8	3.5
Fruit and vegetables	2839	613/1707	35.9	4.1	3.5
Meat and meat products	1286	291/997	29.2	3.1	2.4
Non-alcoholic beverages	1983	415/1406	29.5	4.3	2.5
Sauces, dressings, spreads and dips	1810	249/1756	14.2	3.3	2.5
Snackfoods	480	85/465	18.3	3.0	2.6
Sugars, honey and related products	309	39/283	13.8	1.0	1.3
Special foods (fitness and diet products)	648	56/286	19.6	4.4	3.5
Excluded categories	5128				
Total	23,859	4348/15,767	27.6	3.4	2.7

**Table 2:** HSR uptake by manufacturer, and mean HSR by manufacturer among products with a HSR logo on pack, and those either not displaying the HSR logo or carrying the energy icon only in the 2017 FoodSwitch Monitoring Dataset.

Manufacturer	Products Surveyed	Displaying HSR/Eligible to Display HSR	Displaying HSR	Mean HSR	
	*N*	*n*/*N*	%	HSR Logo on Pack	No HSR/Energy Icon Only
Coles Supermarkets Australia Pty Ltd.	1873	1246/1450	85.9	3.0	3.0
Australian Health & Nutrition Association	115	96/115	83.5	4.2	3.7
Woolworths	1269	713/936	76.2	3.2	3.6
Coca Cola Amatil Ltd.	195	130/171	76.0	3.5	2.1
Simplot Australia (Holdings) Pty Ltd.	415	280/415	67.5	4.0	3.5
Nestle Australia Ltd.	412	218/330	66.1	4.0	1.5
Unilever Australia Pty Ltd.	289	99/245	40.4	3.3	1.9
Campbell Arnott’s	224	83/224	37.1	2.5	2.5
Lion Pty Ltd.	380	97/266	36.5	4.2	2.8
Aldi	1592	428/1369	31.3	3.5	2.6
George Weston Foods Ltd.	155	40/155	25.8	3.9	2.4
Mars Australia	421	54/265	20.4	3.5	2.3
Heinz Australia	325	53/264	20.1	3.9	3.0
Schweppes Australia Pty Ltd.	141	26/139	18.7	NA	1.9
McCain Foods Aust Pty Ltd.	110	14/110	12.7	3.8	3.0
Parmalat Australia Ltd.	164	9/155	5.8	4.1	3.3
Monde Nissin (Australia) Pty Ltd.	229	13/228	5.7	3.5	3.2
Goodman Fielder Ltd.	207	5/191	2.6	4.4	2.6
Manassen Foods Australia Pty Ltd.	201	4/186	2.2	3.9	3.0
Ricegrowers Ltd.	151	3/144	2.1	4.0	3.2
IGA	241	1/151	0.7	5.0	2.9
Mondelez Australia Pty Ltd.	304	0/304	0.0	NA	1.3
San Remo Macaroni Company Pty Ltd.	167	0/167	0.0	NA	3.2
Oriental Merchant Pty Ltd.	155	0/155	0.0	NA	2.0
PepsiCo ANZ	144	0/144	0.0	NA	3.1
General Mills Australia	129	0/129	0.0	NA	2.9
All other manufacturers (<100 products)	13,851	736/7359	10.0	4.0	2.7
Total	23,859	4348/15,767	27.6	3.4	2.7

## Appendix A

Permitted variants of the Health Star Rating system graphic, extracted from the Health Star Rating Style Guide for Industry (December 2017, Version 5) [17].

Option 1: Health Star Rating + energy icon + 3 prescribed nutrient icon + 1 optional nutrient icon

Option 2 Health Star Rating + energy icon + 3 prescribed nutrient icons

Option 3: Health Star Rating + energy icon

Option 4: Health Star Rating only

Option 5: Energy icon only

For the purposes of our findings, we compared the mean HSR of products displaying the HSR logo (Options 1–4) to those carrying no HSR logo (i.e., displaying the Option 5 energy icon only or no HSR variant at all).

## Appendix B

Manufacturers (listed alphabetically) using the HSR system on at least one product in the 2017 FoodSwitch Monitoring Database. One asterisk (*) is used to denote manufacturers using the HSR logo on ≥50% of products; two asterisks (**) for those displaying the HSR logo on ≥75% of products captured.

**Manufacturer (A–Z)**			
Aldi	Goodman Fielder Ltd.	PM Fresh Pty Ltd. **	Unilever Australia Pty Ltd.
Anchor Foods	Greencore Group plc **	Popina (Vic) Pty Ltd. **	Unistraw International Limited **
Andrews Meat Industries **	Green’s General Foods Pty Ltd.	Prolife Foods Ltd.	Vitality Brands Worldwide Pty Ltd.
Angeles Fine Foods Australia Pty Ltd.	Grove Fruit Juices Pty Ltd.	PureBred **	Vitasoy Australia Products Pty Ltd. **
Annex Foods Pty Ltd.	Harvest Box Pty Ltd. **	Quality Food World Pty Ltd.	Warrnambool Cheese & Butter Factory Company
Asahi Beverages	HealthFarm Fine Foods	Quinn Foods Pty Ltd.	Weight Watchers International Inc.
Associated British Foods *	Heinz Australia	Real Foods Pty Ltd. **	Woolworths **
Atkins Nutritionals Inc	IGA	Red Bull Australia Pty Ltd.	Yianni Yogurt **
Australian Eatwell Pty Ltd. *	Interlink Foods Ltd. **	Ricegrowers Ltd.	Yoodles Foods Pty Ltd. **
Australian Health & Nutrition Association **	INVO ANZ Pty Ltd. **	Rinoldi Pasta Pty Ltd.	
Beanfields, PBC **	Kalfresh	Rivalea (Australia) Pty Ltd. **	
Bertalli’s Bakery Pty Ltd.	Kellogg Australia Pty Ltd.	Rousche Group Pty Ltd. **	
BH Fine Foods Pty Ltd.	Kez’s Kitchen	Safcol Australia Pty Ltd.	
Body Science International Pty Ltd.	Life Health Food Ltd. **	Salad Fresh Pty Ltd.	
Boost Fruit Juice Pty Ltd. **	Lindt & Sprungli Australia	Sargents Pty Ltd.	
Campbell Arnott’s	Lion Pty Ltd.	Schweppes Australia Pty Ltd.	
Carman’s Fine Foods Pty Ltd.	Macro Meats Pty Ltd.	Sealord Group Ltd.	
Coca Cola Amatil Ltd. **	Manassen Foods Australia Pty Ltd.	Select Harvests Ltd.	
Coco & Lucas’ ™ Kitchen Pty Ltd. **	Marlow Foods Limited **	Simmone Logue Fine Food Company	
Coconut Collective Pty Ltd. **	Mars Australia	Simplot Australia (Holdings) Pty Ltd.	
Coles Supermarkets Australia Pty Ltd. **	McCain Foods Aust Pty Ltd.	Slim Secrets	
Dick Smith Foods Pty Ltd.	Modern Baking Australia Pty Ltd.	Smart Living Nutrition Pty Ltd. **	
D’Lite Food Pty Ltd. **	Monde Nissin (Australia) Pty Ltd.	SPC Ardmona Operations Limited	
Earth’s Bounty Fine Food Inc. **	Monster Muesli **	Stahmann Farms Enterprises Pty Ltd. **	
Emma & Tom Foods Pty Ltd.	Mountain Bread Company	Sunpork Fresh Foods Pty Ltd. **	
FAL Healthy Beverages Pty Ltd. **	Nestle Australia Ltd.	Sunraysia Natural Beverage Co *	
Fine Fettle Foods	Newfresh Foods Pty Ltd. **	Suntory (Aust) Pty Ltd.	
FODMAPPED Foods Pty Ltd. **	Norco Co-operative Limited **	Symington’s Australia Pty Ltd.	
Food For Health Pty Ltd. **	Norfolk Foods Pty Ltd. **	Table of Plenty	
Food Revolution Group Ltd. **	Pacific West Foods *	Tasti Products Ltd.	
Freedom Foods Group Ltd.	Pangkarra Pty Ltd. **	Tegel Foods Ltd. **	
Frucor Beverages Australia Pty Ltd.	Parilla Fresh **	The Chia Company	
Fyna Foods Australia Pty Ltd.	Parmalat Australia Ltd.	The Happy Snack Company **	
George Weston Foods Ltd.	Patties Foods	The Northern Co-operative Meat Company Ltd. **	
Go Natural	Perfection Fresh Australia Pty Ltd. **	The Yoghurt Co Pty Ltd.	
Goodman Fielder Ltd.	Picasso Foods **	Think Products **	
Greencore Group plc **	Picot Productions Ltd. **	Thirsty Brothers Pty Ltd. **	
